# Subsidence of the Cholera in the West

**Published:** 1833

**Authors:** 


### Art. XVIII. Subsidence of the Cholera in the West. Health of Cincinnati.

Since the beginning of Autumn, we have heard of no Epidemic Cholera in the Valley of the Mississippi. It seems to have subsided with our Bilious Fevers. In Cincinnati, for the last two months, we have not heard of a single case, under all the variety of powerful, exciting causes to which our people have exposed themselves. The city, moreover, in reference to other diseases, has been highly favored.

By consulting the bills of mortality for November and December, during a period of five years, we are enabled to institute the following comparison:

| | | | | |
|---|---|---|---|---|
| 1829 | November and | December | 88 | deaths. |
| '30 | do | do | 136 | do. |
| '31 | do | do | 163 | do. |
| '32 | do | do | 139 | do. |
| '33 | do | do | 94 | do. |

The population of the city at the present time, is considerably beyond what it was in 1829, so that the proportional mortality of the months of November and December of this year, is less than it was for the preceding four years. The contrast which it makes with that of 1831, is striking. That was the autumn preceding the appearance of the Cholera among us. Had the atmospheric deterioration then begun, and is the great healthiness of the present period, to be considered as an evidence that the distemperature has *finally* ceased? This is at least a pleasant speculation.

